# Francesco Torti (1658-1741) and his description on the therapeutic use of quinine: the first effective method of treating malaria based on an extract of the bark of the Peruvian cinchona tree

**DOI:** 10.5281/zenodo.20506312

**Published:** 2026-06-02

**Authors:** Marco Artico, Roberta Costi, Valentina Gazzaniga, Silvia Iorio, Francesco Saverio Pastore, Luigi Cofone

**Affiliations:** 1 Department of Sense Organs, University of Rome “La Sapienza”, 00161 Rome, Italy.; 2 Department of Chemistry and Pharmaceutical Technology, University of Rome “La Sapienza”, 00161 Rome, Italy.; 3 Department of Medico-Surgical Sciences and Biotechnologies, “Sapienza” University of Rome, Rome, Italy.; 4 Department of Surgery, University of Nostra Signora del Buon Consiglio, Tirana, Albania.

## Abstract

The work of Francesco Torti (1658–1741), who developed the use of cinchona bark, the source of quinine, to treat malaria, is examined in this paper. Torti was notable for his meticulous patient care and clinical observation during a period when humoral theories still influenced medical practice. He introduced the famous "fever tree" in his 1712 book, which detailed several forms of intermittent fevers and looked at how they responded to therapy. His efforts contributed to the growth of cinchona throughout Europe and is indicative of a larger trend in medicine toward a more patient-centred and experience-based approach.

Malaria was one of the most common and debilitating diseases in Europe, particularly in humid regions with stagnant waters. Common symptoms include frequent episodes of fever accompanied by chills, excessive sweating, headaches, and general malaise. The erythrocytic life cycle of *Plasmodium* parasites in the human host is commonly reflected in the cyclical pattern of fever bouts, which often occur every 48 (tertian malaria) or 72 hrs (quartan malaria) [1, 2].

However, it is crucial to remember that early modern doctors used a broad and adaptable term of ‘fevers’, which included a variety of ailments, rather than the contemporary understanding of malaria as a parasite illness. Because ‘fever’, ‘intermittent fever’, and what is now known as malaria only partially overlapped, care must be taken when retrospectively aligning early modern illness categories with contemporary biomedical terminology [3]. Classical humoral medicine did not understand the nature of intermittent fevers, and treatments such as bloodletting, purging and dietary management were often used, despite their ineffectiveness. Although these treatments were widely accepted within the Galenic tradition, they often proved ineffective against malaria [4]. Fever theory throughout the 17^th^ and early 18^th^ centuries, however, was neither consistent nor permanent. Medical reasoning was still influenced by Galenic humoral presumptions, but they were being questioned and reinterpreted in light of new theories such as Cartesian mechanism and Harveian circulation theory. Consequently, classic and novel explanatory models coexisted in medical understandings of fever [5].

A major shift in the treatment of these fevers occurred throughout the 17^th^ and early 18^th^ century with the gradual introduction of cinchona bark, a substance made from trees native to the Andean region of South America. One of the doctors that made a substantial contribution to proving the medicinal benefits of cinchona bark as a dependable treatment for sporadic fevers was Francesco Torti [2,6,7].

Cinchona bark's entry into European medicine should be viewed as part of a larger, uneven process of commercialisation rather than as a singular medicinal breakthrough. The drug first made its way into the medical industry via missionary and colonial networks, where it was marketed as a novel and frequently misunderstood cure. A protracted period of testing and discussion ensued, during which doctors, patients, and traders all added to the body of useful knowledge [3]. Cinchona did not stabilise as an accepted and standardised treatment in pharmaceutical and medical practice until much later [5]. Torti's work can be positioned within this intermediate stage of consolidation rather than at the beginning of the process within this larger trajectory.

Torti, who was born in Modena in 1658, attended the University of Bologna to study medicine and graduated in 1678. He returned to Modena after completing his studies, where he worked as a physician for the Este court and as a medical professor. As a physician, he was highly regarded by his colleagues, and later medical historians have often emphasised the importance he attached to direct clinical observation. For this reason, he was nicknamed ‘the Hippocrates of Modena’ for his empirical approach, which reflected both his pragmatic approach to medicine and his commitment to careful observation of patients [8].

Throughout Torti's lifetime, intermittent fevers were widespread throughout Europe, and even though the parasite cause of malaria would not be discovered until the late 19^th^ century, doctors were fully aware of the characteristic cyclical pattern of these fevers. The widely accepted view connected these diseases to environmental factors, particularly the effects of standing water and miasmas from marshes [1, 2].

The use of cinchona bark in European medicine represented an important turning point in this field, as its medicinal properties, already known to indigenous Andean cultures, who had long used medicines derived from the cinchona tree, made it possible to treat feverish illnesses. Spanish colonial networks and Jesuit missionaries contributed to the dissemination of this remedy throughout Europe in the 17^th^ century. The substance soon acquired a number of names, including ‘Peruvian bark’ and ‘Jesuit's powder’, which represented both its source and the ways in which it had been introduced into European medicine [6, 9].

Cinchona bark's spread was directly related to its use in a growing medical industry. Increasingly, remedies were marketed through written media like newspapers and delivered through commercial networks as commodities. By highlighting specific applications, doses, and indications, these procedures shaped medical knowledge while also helping to stabilise the bark as a recognisable product [5].

Despite increasing reports of successful treatments, cinchona bark first encountered a great deal of skepticism among European physicians. Many practitioners were hesitant to utilise the treatment because its therapeutic benefits looked difficult to reconcile with the prevalent ideas of humoral medicine. The cornerstone of conventional therapy was the removal of purportedly harmful humors using evacuative methods like bloodletting or purging. In contrast, cinchona bark was found to be able to stop the fever cycle without having such evacuative effects. This seeming contradiction sparked ongoing debates over how best to use it [2, 4]. Because cinchona's therapeutic benefits did not readily fit with accepted humoral explanations, they further exacerbated conflicts within preexisting medical frameworks. Its use coexisted with earlier theories and helped to gradually reconfigure them rather than instantly replacing them [5].

Torti’s groundbreaking publication, *Therapeutice specialis ad Febres quasdam Perniciosas, inopinatò, ac repenté lethales, una verò China China, peculiari Methodo ministrata, sanabiles*, which focused on the study and treatment of severe fever illnesses, was released in 1712. Torti's work differed from many earlier medical treatises due to its emphasis on clinical observation. Rather than relying mostly on theoretical speculation, he grounded his research on careful patient observation and the beneficial results of therapeutic techniques [10, 11].

A fundamental became diagnostic criteria. This perspective reflected the broader shift in early modern medicine toward empirical reasoning and represented a significant methodological leap [6]. However, this shift should not be interpreted as merely a step towards modern therapeutic reasoning. Rather, Torti's work should be seen as a component of the ongoing evolution of medical knowledge, which reflects the cohabitation of inherited theoretical frameworks and practical practices [5].

One of Torti's most well-known illustrations is the "tree of fevers," or Lignum febrium (Figure 1). In this schematic illustration, the different febrile states were categorised according to their periodicity and response to cinchona bark. Intermittent fevers, particularly tertian and quartan fevers, were shown to be very responsive to the treatment, although continuous fevers generally did not [1, 2].

**Figure 1:**
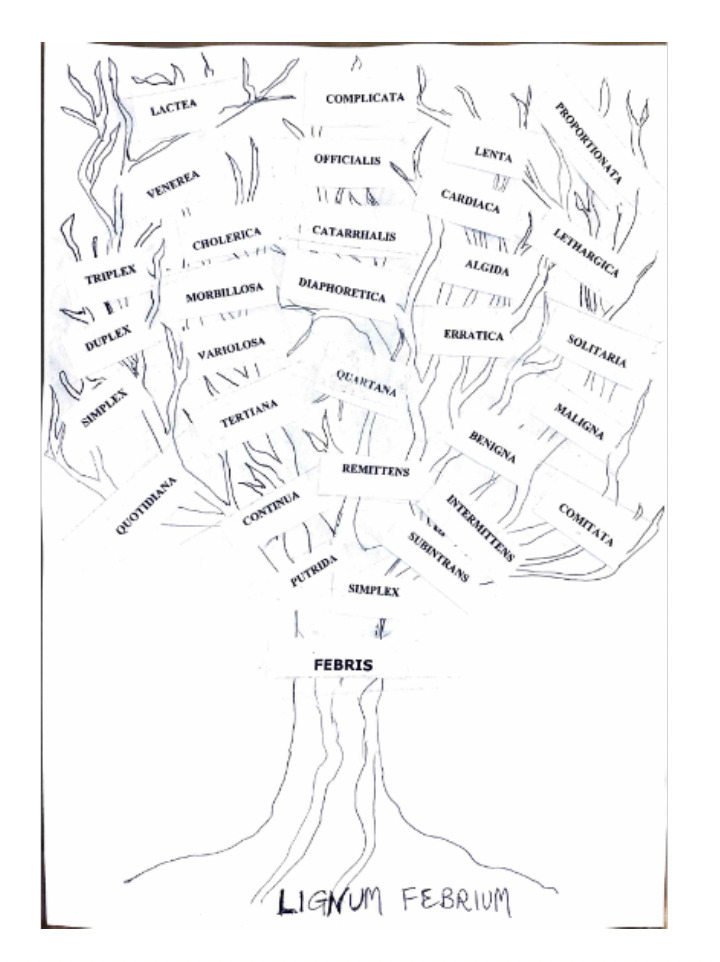
The fever tree according to Francesco Torti, freely redrawn and reinterpreted by Professor M. Artico (by kind courtesy of the author).

Torti's *Lignum febrium* should be seen as an ambitious attempt to arrange a very complicated and unstable disease category into a logical visual system rather than as a straightforward classification tool. The figure, which represented the variety of intermittent and continuous fevers experienced in actuality, was much more complex in its original form than contemporary simplified copies imply. This intricacy links classification to therapy response and emphasises the ongoing challenges in characterising febrile illnesses [5].

Torti also emphasised the importance of administering cinchona bark at the appropriate time throughout the illness. Based on his professional experience, he recommended that the drug be given in between fever episodes instead of during the acute febrile episode. Ignoring these therapeutic indications could lead to treatment failure, which also likely contributed to some physicians' doubts about the effectiveness of the treatment, especially Bernardino Ramazzini [6].

Torti's research played a significant role in the acceptance of cinchona bark as the best cure for intermittent fevers, despite the opinions of his colleagues. The drug spread throughout Europe and remained the most widely used treatment for malaria for over a century. A scientific explanation for the therapeutic benefits observed in clinical practice only came in 1820, when the active alkaloid quinine was isolated [6, 12]).

Torti’s work represents an important development in the history of medical thought. His emphasis on therapeutic experimentation, careful clinical observation, and the evaluation of outcomes reflects the broader transformations that characterised medicine in the early modern period. Through his systematic study of the effects of cinchona bark on intermittent fevers, Torti contributed to the development of one of the earliest effective treatments for what would later be identified as malaria. At the same time, his approach helped to promote a more empirical orientation in medical practice, grounded in observation and experience rather than purely theoretical speculative reasoning. Another element of Torti's approach was the idea that different types of fever might be distinguished by their responses to cinchona bark; he recommended that physicians consider whether a particular fever responded to treatment with Peruvian bark. The therapeutic effect of the medication thus became a crucial diagnostic criterion that helped doctors differentiate between feverish conditions that responded to cinchona bark and those that did not.
